# Dynamic changes in fungal communities and functions in different air-curing stages of cigar tobacco leaves

**DOI:** 10.3389/fmicb.2024.1361649

**Published:** 2024-03-19

**Authors:** Songchao Zhao, Yuanyuan Li, Fang Liu, Zhaopeng Song, Weili Yang, Yunkang Lei, Pei Tian, Mingqin Zhao

**Affiliations:** ^1^College of Tobacco Science, Flavors and Fragrance Engineering and Technology Research Center of Henan Province, Henan Agricultural University, Zhengzhou, Henan, China; ^2^Dazhou City Branch of Sichuan Province Tobacco Company, Dazhou, Sichuan, China; ^3^Deyang City Branch of Sichuan Province Tobacco Company, Deyang, Sichaun, China; ^4^China Tobacco Jiangshu Industry Co., Ltd., Xuzhou Cigarette Factory, Xuzhou, Jiangsu, China

**Keywords:** air curing, chemical composition, fungal community, predicted function, tobacco leaf, association analysis

## Abstract

**Introduction:**

Air curing (AC) plays a crucial role in cigar tobacco leaf production. The AC environment is relatively mild, contributing to a diverse microbiome. Fungi are important components of the tobacco and environmental microbiota. However, our understanding of the composition and function of fungal communities in AC remains limited.

**Methods:**

In this study, changes in the chemical constituents and fungal community composition of cigar tobacco leaves during AC were evaluated using flow analysis and high-throughput sequencing.

**Results:**

The moisture, water-soluble sugar, starch, total nitrogen, and protein contents of tobacco leaves exhibited decreasing trends, whereas nicotine showed an initial increase, followed by a decline. As determined by high-throughput sequencing, fungal taxa differed among all stages of AC. Functional prediction showed that saprophytic fungi were the most prevalent type during the AC process and that the chemical composition of tobacco leaves is significantly correlated with saprophytic fungi.

**Conclusion:**

This study provides a deeper understanding of the dynamic changes in fungal communities during the AC process in cigar tobacco leaves and offers theoretical guidance for the application of microorganisms during the AC process.

## 1 Introduction

After mature harvesting, tobacco leaves require further processing to transform them from an unsmokable to a smokable state. This process is known as air curing (AC) and is a critical step in tobacco production. Unlike tobacco leaves for cigarettes, which are flue-cured, tobacco leaves for cigars are air-cured; this difference in the AC process accounts for the unique flavor and aromatic characteristics of cigars (Wang et al., 2009; Sun et al., 2019; Liu H. et al., 2021; Liu F. et al., 2021). The AC of cigar tobacco leaves refers to the process of gradually drying fresh tobacco leaves under natural or controlled temperature, humidity, and ventilation conditions, causing changes in the internal substances of the leaves to achieve the desired quality. Therefore, the AC of cigar tobacco leaves is not simply a matter of drying the leaves but involves delicate coordination between the chemical composition of the leaves and drying speed (Salehzade et al., 2009; He et al., 2020). The entire AC process can be divided into four distinct stages: wilting, yellowing, browning, and stem-drying. During AC, there are significant changes in water-soluble sugar, starch, total nitrogen, nicotine, and proteins (Lu et al., 2020; Liu F. et al., 2021; Liu H. et al., 2021). Because these indicators have a significant impact on the quality of tobacco leaves (e.g., nicotine plays a decisive role in sensory intensity; Yildiz, 2004; Liu F. et al., 2021; Liu H. et al., 2021; Zhao et al., 2022), they have been a focus of research. Some studies have demonstrated that fungal community structure and its function are closely related to tobacco quality; however, fungal community structure and function in the AC process of cigar tobacco and their relationship with tobacco quality are not clear (Su et al., 2022; Wang et al., 2022).

During the growth of tobacco in the field, substantial metabolic products are excreted or leaked from the leaves, providing abundant nutrition for the growth of various microorganisms (Capdesuñer et al., 2019; Brescia et al., 2020). Consequently, a unique microenvironment is formed on the surface and inside of mature harvested tobacco leaves, where a substantial number of microorganisms exist. After harvesting, the mild conditions of the AC process for cigar tobacco leaves allow for the presence of abundant microorganisms both inside and on the surface of the leaves (Zhao et al., 2019; Koskella, 2020). Fungi are a major group of microorganisms with crucial roles in the natural world. Mycorrhizal and endophytic fungi can enhance plant resistance to diseases and promote plant growth (Yan et al., 2019; Huang et al., 2020; Li et al., 2023). Wang et al. (2023) found that the ectomycorrhizal and saprotrophic communities changed with change in successional stage as the number of years increased by studying changes in functional fungi during forest succession. For example, the relative abundance of saprotrophic fungi increased. Fungi also play an important role in tobacco production. Based on a sequencing analysis of the leaves of cured tobacco during flue curing, Chen Y. et al. (2020) found that *Alternaria* is always rich and the proportion of saprotrophs increased with the prolongation of flue curing time. Of note, saprotrophic fungi are often used to degrade macromolecules in tobacco leaves to reduce the irritancy produced during combustion and enhance the aromatic quality (Gao et al., 2012; Su et al., 2016). Hopkins et al. (2018) found a high frequency of saprophytic taxa among soil fungi affected by drought. The AC of cigar tobacco leaves, on the other hand, involves gradual water loss under the influence of temperature and humidity, equivalent to changes in tobacco leaves under arid conditions. However, the effects of drying on fungi in cigar tobacco leaves and the functional groups of fungi remain unclear. Some studies have evaluated the correlations between microorganisms and crop quality during the curing process (Uzoma et al., 2024), including the curing of tobacco leaves (Chen Q. L. et al., 2020; Hu et al., 2021; Zhang et al., 2023). However, reports on fungal community structure and fungal function in the AC process of cigar tobacco leaves are lacking. To understand the succession and function of the fungal community, which could facilitate rational regulation of fungal community structure and function for the production of higher-quality cigar tobacco during the later AC period, the dynamic changes in structure and function of fungi during the AC of cigar tobacco leaves were analyzed in the present study.

To gain insights into the changes in fungal communities during the drying process of cigar tobacco and the influence of fungal functions on tobacco quality, we employed ITS sequencing technology and the Illumina platform to evaluate fungi during the AC process of cigar tobacco, predict fungal functions during AC, and determine the correlations between the functions of fungi and key quality indicators of cigar tobacco during AC. Finally, we aimed to clarify the role of fungi during the AC process of cigar tobacco.

## 2 Materials and methods

### 2.1 Experimental materials

This experiment was conducted in 2022 in Dazhou City, Sichuan Province, China (107°97′E, 31°51′N). The soil texture in the experimental area was sandy loam with an available nitrogen content of 92 mg/kg, available phosphorus content of 8.8 mg/kg, available potassium content of 114.0 mg/kg, and a pH value of 5.6. Chuanxue No. 2 is the main variety planted in the area. Therefore, Chuanxue No. 2 was selected as the test variety.

### 2.2 Sample collection

**Figure 1:**
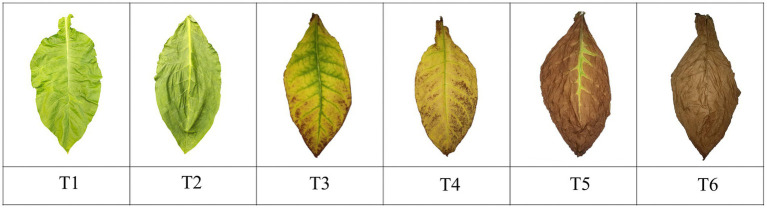
Changes in cigar tobacco leaves during the AC process. T1, day 1; T2, withering period, day 3; T3, yellowing period, day 6; T4, browning period, day 14; T5, stem-AC period, day 21; T6, end of AC, day 30.

The Chuanxue 2 variety was transplanted on 6 May 2022. Tobacco plants were selected for harvesting at essentially the same leaf maturity stage; tobacco leaves were yellow-green in appearance, with 4/5 of the main veins turning white to all white, 2/3 of tertiary veins turning white, and maturation spots beginning to appear. The experimental material was the middle leaves (11^th^–12^th^ leaves), which were picked and hung in a chamber with constant temperature and humidity. Tobacco leaves were collected at different stages during the AC process. The specific stages and times were fresh tobacco T1 (day of harvest), withering period T2 (day 3 of AC), yellowing period T3 (day 6 of AC), browning period T4 (day 14 of AC), stem-AC period T5 (day 21 of AC), and end of AC T6 (day 30 of AC), with each sample collected between 8:00 and 9:00 a.m. The color and condition of the tobacco at the time of sampling are shown in Figure 1. At each sampling time, six tobacco leaves with similar appearances were randomly selected to remove the main vein and branch vein and divided into three parts: one part was used to determine the moisture content of the tobacco leaves, another part was wrapped in tin foil and stored at −80°C for analyses of the tobacco leaf microbiota, and the final part was dried and the water-soluble sugar, starch, total nitrogen, nicotine, and protein contents of the tobacco leaves were measured after removal of the main vein. All tests were performed in quadruplicate (Wang et al., 2024). Specific details of the temperature and humidity designs are listed in Table 1.

**Table 1:** Temperature and humidity conditions for different periods during AC.

	Wilting stage	Yellowing stage	Browning stage	Stem-drying stage
Humidity	80%	75%	70%	45%
Temperature	22°C	26°C	30°C	35°C

### 2.3 Tobacco leaf moisture content

The tobacco leaves were first weighed to obtain weight “a.” The tobacco was then dried in an oven until the weight remained the same and then weighed again to obtain weight “b.” Test treatments were performed in quadruplicate. The weights were used to obtain the moisture content as follows:

$$\mathrm{moisture}\;\mathrm{content}\;\left(\%\right)=\frac{a-b}{a}\times 100$$

where a is the initial weight and b is the weight after drying.

### 2.4 Indicators of tobacco leaf quality

Tobacco leaf samples taken at each AC stage were dried and ground to pass through a 60-mesh sieve. Afterward, the samples (0.25 g) were weighed and placed in 50 mL plastic bottles. Then, 25 mL of 5% acetic acid solution was added, and the mixture was shaken for 30 min. Next, the mixture was filtered using quantitative filter paper, and the filtrate was assayed for water-soluble sugar, starch, total nitrogen, nicotine, and protein content (Cheng et al., 2011). The test treatments were performed in quadruplicate.

### 2.5 DNA extraction and sequencing

Tobacco (1 g) was ground in liquid nitrogen. A 20-mg sample was obtained from the ground sample and the DNA of the sample was extracted using the E.Z.N.A.® Soil DNA Kit (Omega Bio-tek, Norcross, GA, United States) kit according to the manufacturer’s instructions (Ye et al., 2017). After DNA extraction, 1% agarose gel electrophoresis was used to detect the quality of the extracted DNA, and the NanoDrop2000 (Thermo Fisher Scientific Inc., Waltham, MA, United States) was used to measure the DNA concentration and purity. Subsequently, PCR was used to amplify the variable region of the fungal ITS region ITS1 using the primer sequences ITS1F (5′-CTTGGTCATTTAGAGGAAGTAA-3′) and ITS2R (5′-GCTGCGTTCTTCATCGATGC-3′; Liu H. et al., 2021; Liu F. et al., 2021). Subsequently, the PCR products from the same sample were mixed and recovered using a 2% agarose gel. An AxyPrep DNA Gel Extraction Kit (Axygen Biosciences, Union City, CA, United States) was used to purify the recovered products according to the manufacturer’s instructions, followed by 2% agarose gel electrophoresis to check the purity. The recovered products were quantified using a Quantus™ Fluorometer (Promega, Co., Madison, WI, United States), and library preparation was carried out using a NEXTflex Rapid DNA-Seq Kit. Sequencing was performed using an Illumina MiSeq PE300 platform.

### 2.6 Processing of sequencing data

Raw sequencing reads were subjected to quality control and merging using fastp (Chen et al., 2018; https://github.com/OpenGene/fastp, version 0.20.0) and FLASH (Magoč and Salzberg, 2011; http://www.cbcb.umd.edu/software/flash, version 1.2.7). Subsequently, high-quality and merged sequences were denoised using the DADA2 (Callahan et al., 2016) plugin in the Qiime2 workflow (Bolyen et al., 2019). Denoised sequences obtained using DADA2 are referred to as amplicon sequence variants (ASVs). Taxonomic classification of ASVs was performed using the naïve Bayes classifier implemented in Qiime2 based on the UNITE database (v 8.99).

The fungal community of cigar tobacco leaves was analyzed using QIIME2 (2020.2 release), and 2,704,932 fungal sequence reads were obtained from all cigar tobacco leaf samples. To account for potential bias resulting from uneven sequencing depth among the samples, the sequencing count for each sample was rarefied to the depth of the sample with the lowest read count. Additionally, ASVs that accounted for less than 0.01% of the total sequences across all the samples were removed. In total, 86,250 effective fungal sequences were retained for subsequent analyses. The sequence analysis revealed 175 fungal ASVs in all samples.

### 2.7 Data analysis

Chemical composition data for the cigar tobacco leaves during the AC process were plotted using Microsoft Excel 2016. One-way ANOVA was performed using SPSS 21.0 to determine if there were significant differences (*p* < 0.05) between groups. Alpha-diversity indices, community composition analysis, functional predictions, and a difference analysis of fungal genera (one-way ANOVA, FDR multiple testing, and Tukey–Kramer tests with a threshold probability of 0.95) were conducted using the Majorbio Cloud Platform (Qiime2 workflow)^1^ and the FUNGuild database.^2^ The constrained principal coordinate analysis (CPCoA), ANOSIM, and correlation analyses of fungi with chemical composition were performed using the Lingbo MicroClass platform.^3^ In addition, all fungal genera in tobacco were tested using Spearman correlation analyses (*p* < 0.05) and a fungal network was visualized using Gephi (version 0.9.7).

## 3 Results

### 3.1 Changes in moisture content and quality indicators of cigar tobacco during AC

**Figure 2:**
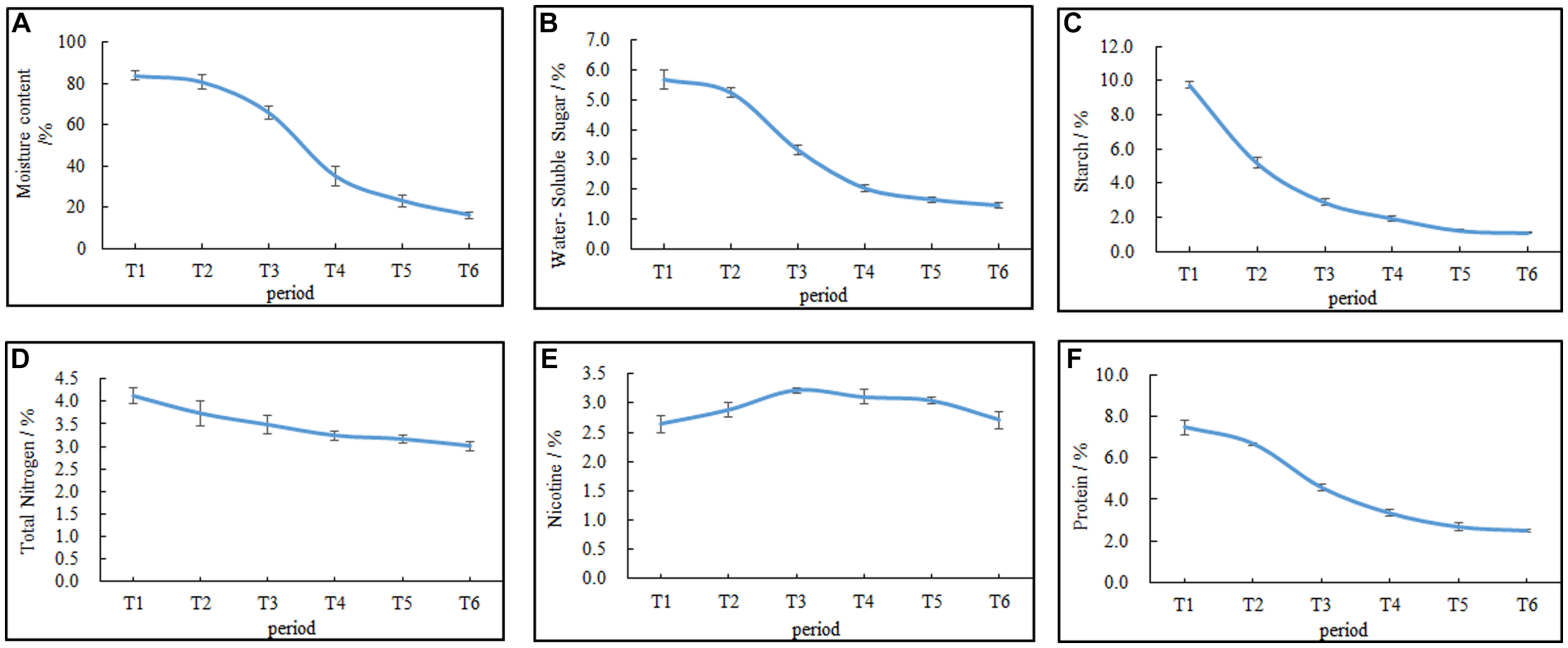
Changes in the moisture content and chemical composition during AC of cigar tobacco leaves. **(A)** Moisture content. **(B)** Water-soluble sugar. **(C)** Starch. **(D)** Total nitrogen. **(E)** Nicotine. **(F)** Proteins. Data are presented as the mean ± SD.

The moisture content of the cigar tobacco leaves during AC is shown in Figure 2A. The rate of water loss from the leaves followed an “S”-shaped trend, with a slow initial change and gradual acceleration as the AC process progressed. During the initial phase from T1 (fresh leaves) to T2 (wilting phase), the moisture content of the leaves decreased slowly. However, after T2, the moisture content of the leaves declined rapidly. The differences in moisture content among T3 (yellowing phase), T4 (browning phase), T5 (stem-drying phase), and T6 (end of AC) were all statistically significant (*p* < 0.05), with the moisture content at T6 being 16%, representing an 80.95% decrease compared with that at T1.

Changes in the contents of carbon–nitrogen substances, such as water-soluble sugar, starch, total nitrogen, nicotine, and proteins, during the AC process of cigar tobacco leaves are shown in Figures 2B–F. The water-soluble sugar, total nitrogen, proteins, and starch always decreased during the AC process, and the rate of decrease was faster in the early stages and tended to be slower in the later stages. By the end of the AC process, the water-soluble sugar, total nitrogen, protein, and starch contents decreased by 74.47%, 26.94%, 66.44%, and 91.43%, respectively, with a greater reduction in water-soluble sugar, proteins, and starch than in other components. Nicotine, on the other hand, initially increased and then decreased due to the respiratory loss of dry matter, causing a relative increase in its content, followed by degradation (Lu et al., 2020).

### 3.2 Changes in the community structure of microorganisms during AC

**Figure 3:**
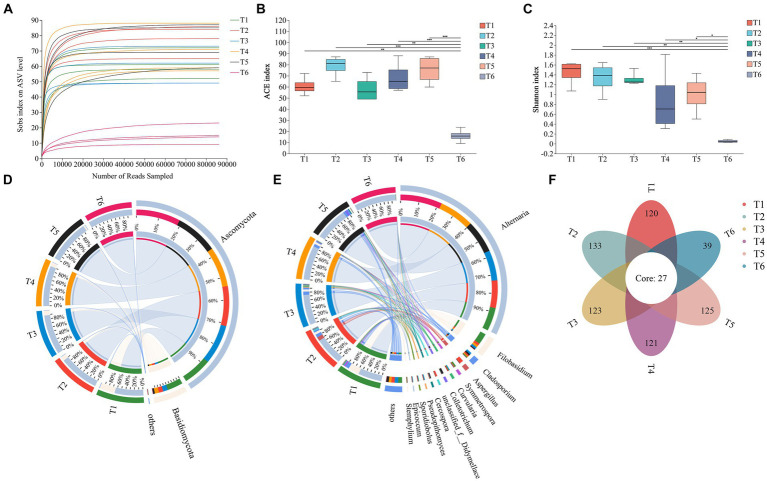
Community structure of fungi. **(A)** Rarefaction curve of ASVs in cigar tobacco leaves during the AC process. **(B)** Fungal ACE index. **(C)** Fungal Shannon index. **(D)** Relative abundances of fungal phyla. **(E)** Relative abundances of fungal genera. **(F)** Numbers of shared and unique ASVs in different AC stages. ^*^p < 0.05, ^**^p < 0.01, and ^***^p < 0.001.

As shown in Figure 3A, as the number of ASVs increased, the rarefaction curve for each sample flattened, indicating that the detection rate of the fungal community in cigar tobacco leaves approached saturation and the sequencing data covered most of the fungal community in the leaves. An alpha-diversity analysis of the fungal community was performed using the ACE index (Figure 3B) to reflect community richness and the Shannon index (Figure 3C) to reflect community diversity. During the T1–T5 AC periods, there were some differences in the richness and diversity of the fungal communities in the leaves; however, these differences were not significant. However, at the end of the AC process (T6), both the richness and diversity of the fungal community in the cigar tobacco leaves decreased.

The microbial sequences of cigar tobacco leaves during the AC process were classified from phylum to species levels using Qiime. At the phylum level (Figure 3D), Ascomycota and Basidiomycota were the most abundant. Other phyla were also detected; however, their abundances were low (<1%). Ascomycota had a relatively high proportion in each period and increased gradually during AC, reaching 99.95% at T6, and was the dominant phylum throughout the AC process. In contrast, Basidiomycota showed a decreasing trend during AC and had the lowest proportion at T6, accounting for only 0.05%. At the genus level (Figure 3E), *Alternaria* consistently showed high abundance throughout all AC stages, and its proportion gradually increased during the tobacco leaf AC process from 56.22% at T1 to 99.63% at T6. *Filobasidium* showed a relatively high proportion of 26.65% at T1 but sharply declined after this stage. Other abundant genera, such as *Aspergillus* (9.19%) at T2, *Cladosporium* (4.40%) at T3, and *Colletotrichum* (4.73%) at T5, dropped to below 1% at T6. A Venn diagram illustrates the shared and unique ASVs during different stages of the cigar tobacco leaf AC process (Figure 3F), with 27 ASVs present in all stages.

### 3.3 Comparative analysis of fungal communities during the AC process

**Figure 4:**
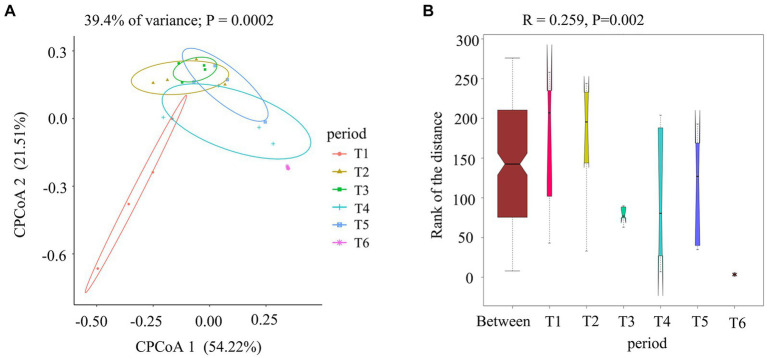
Comparison of fungal communities at different stages of the cigar tobacco AC process. **(A)** Constrained principal coordinate analysis (CPCoA) of fungal communities based on Bray–Curtis distances at different stages. **(B)** ANOSIM of fungal communities.

To gain a visual understanding of the impact of the AC process on fungal communities in cigar tobacco leaves, we compared fungi at different stages of the AC process using CPCoA based on Bray–Curtis distances (Figure 4A) and ANOSIM (Figure 4B). As shown in Figure 4A, the contribution rates of CPCoA axis 1 and CPCoA axis 2 in the process of cigar tobacco AC were 54.22 and 21.51%, respectively, with a cumulative contribution rate of 75.73%. The fresh tobacco leaf (T1) treatment and dry tobacco leaf (T6) treatment at the end of the AC process were significantly different from those in the other treatments. The communities at the wilted period (T2), yellowing period (T3), browning period (T4), and dry-stem period (T5) were relatively close in distance; however, all exhibited significant differences from T1 and T6. The results indicated that the fungal community in cigar tobacco undergoes significant changes after the AC process, which may be related to the abundance of *Alternaria* in the T1 and T6 treatments. *Alternaria* has a strong adaptability to the environment and can dominate even in low-moisture tobacco leaves (Torres et al., 2003). ANOSIM was performed on all samples (*R* = 0.259 > 0, *p* = 0.002 < 0.01), indicating that the differences among the six treatments during the AC process were greater than those within each treatment and were statistically significant.

### 3.4 Impact of the AC process on fungal functions in cigar tobacco leaves

**Figure 5:**
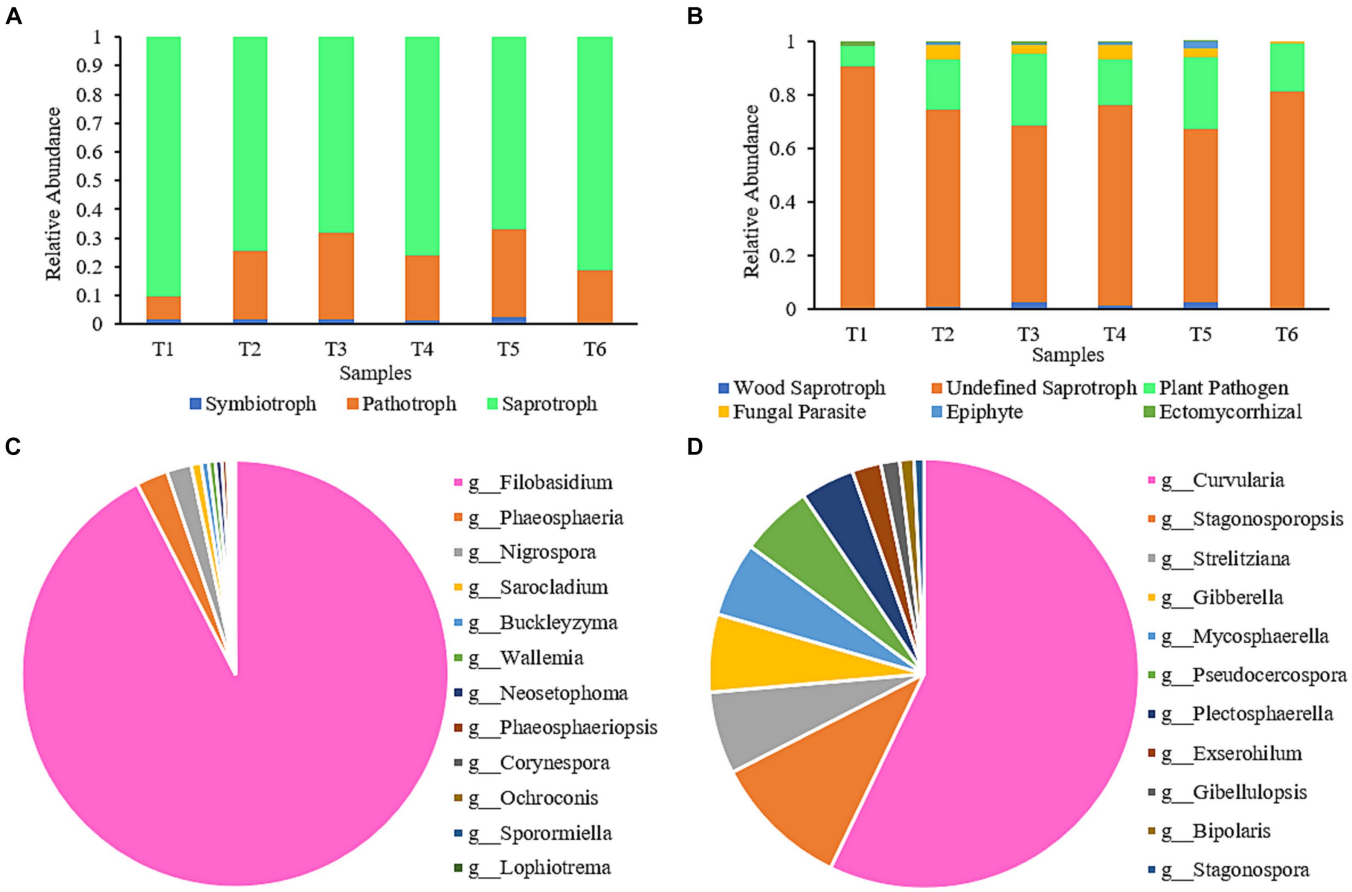
Composition of functional communities during the AC process of cigar tobacco. **(A)** Composition of nutritional modes of fungal communities at different stages; **(B)** Functional group composition of fungal communities at different stages; **(C)** Contribution of fungi in the Undefined Saprotroph group; **(D)** Contribution of fungi in the Plant Pathogen group.

The impact of the AC process on the functional diversity of fungal communities in cigar tobacco leaves was assessed using FUNGuild software. The predicted fungal communities displayed various functional guilds; however, only those identified as having unique functions in FUNGuild and categorized as “highly probable” or “probable” were selected for the analysis (Figure 5A). Fungal functional guilds were classified and analyzed (Figure 5B). The relative abundance of each nutritional mode and functional guild was calculated as the sum of the relative abundances of all ASVs in the particular functional group. As shown in Figure 5A, the fungi in the AC process had three nutritional functions: saprotroph, pathotroph, and symbiotroph. During the AC process, the proportion of the Undefined Saprotroph functional group was highest, followed by Plant Pathogens. These two functional groups belong to the saprotroph and pathotroph nutritional types, respectively. By analyzing the contribution of fungi in the Undefined Saprotroph (Figure 5C) and Plant Pathogen (Figure 5D) functional groups, it can be seen that *Filobasidium* was the predominant genus in the Undefined Saprotroph group, accounting for 92.42%. However, the abundance of *Filobasidium* decreased gradually during the AC process and was only 0.04% at the end of AC. *Curvularia* was the predominant genus in the Plant Pathogen group (Liu et al., 2009), accounting for 57.15%; however, it was a low-abundance genus throughout the AC process and almost disappeared by the end of AC (<0.05%).

### 3.5 Correlations between tobacco quality indicators and fungal functions

**Figure 6:**
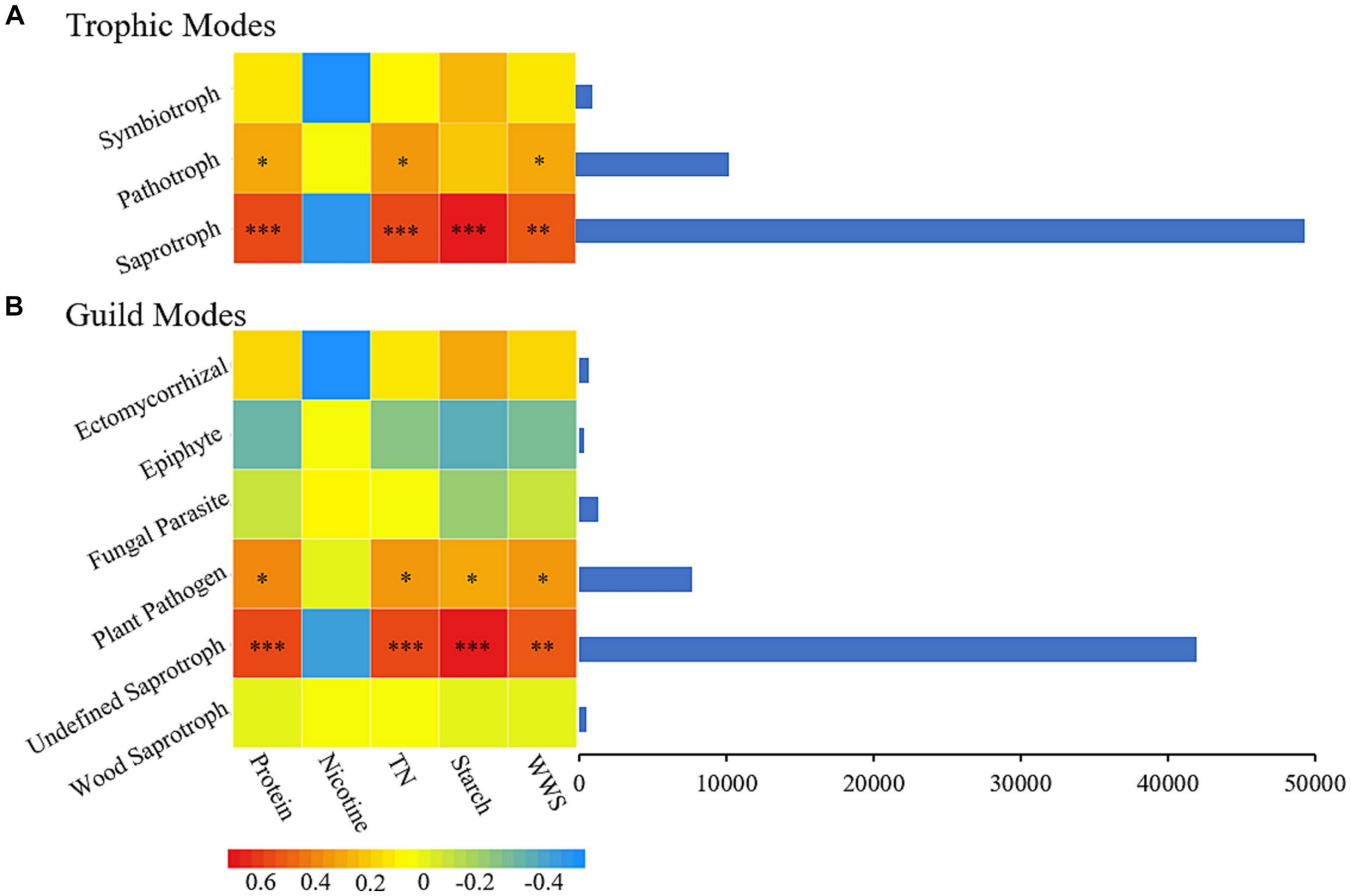
The chemical composition of cigar tobacco is closely related to its fungal nutritional patterns **(A)** and functional groups **(B)**. TN, Total nitrogen; WSS, Water-soluble sugar.

During plant aging, changes in plant material affect the composition of microorganisms, and the dynamic changes in microorganisms, in turn, affect the material properties inside the plant, which promotes plant aging (Verma et al., 2017; Lin et al., 2022). In the process of secondary aging during the AC of tobacco leaves, the correlation between the nutritional mode (Figure 6A) and functional groups (Figure 6B) of fungi and the important carbon and nitrogen substances in tobacco leaves were analyzed. The saprotroph nutritional mode was closely related to four important carbon and nitrogen substances in tobacco leaves, with significant positive correlations with protein, total nitrogen, starch, and water-soluble sugar and no significant correlation with nicotine. Undefined Saprotroph, as an important functional group in the saprotroph nutritional mode, also showed significant correlations (*P*<0.05) with the same four important carbon and nitrogen substances in tobacco leaves.

### 3.6 Analysis of differential fungi and fungal co-occurrence networks during AC

**Figure 7:**
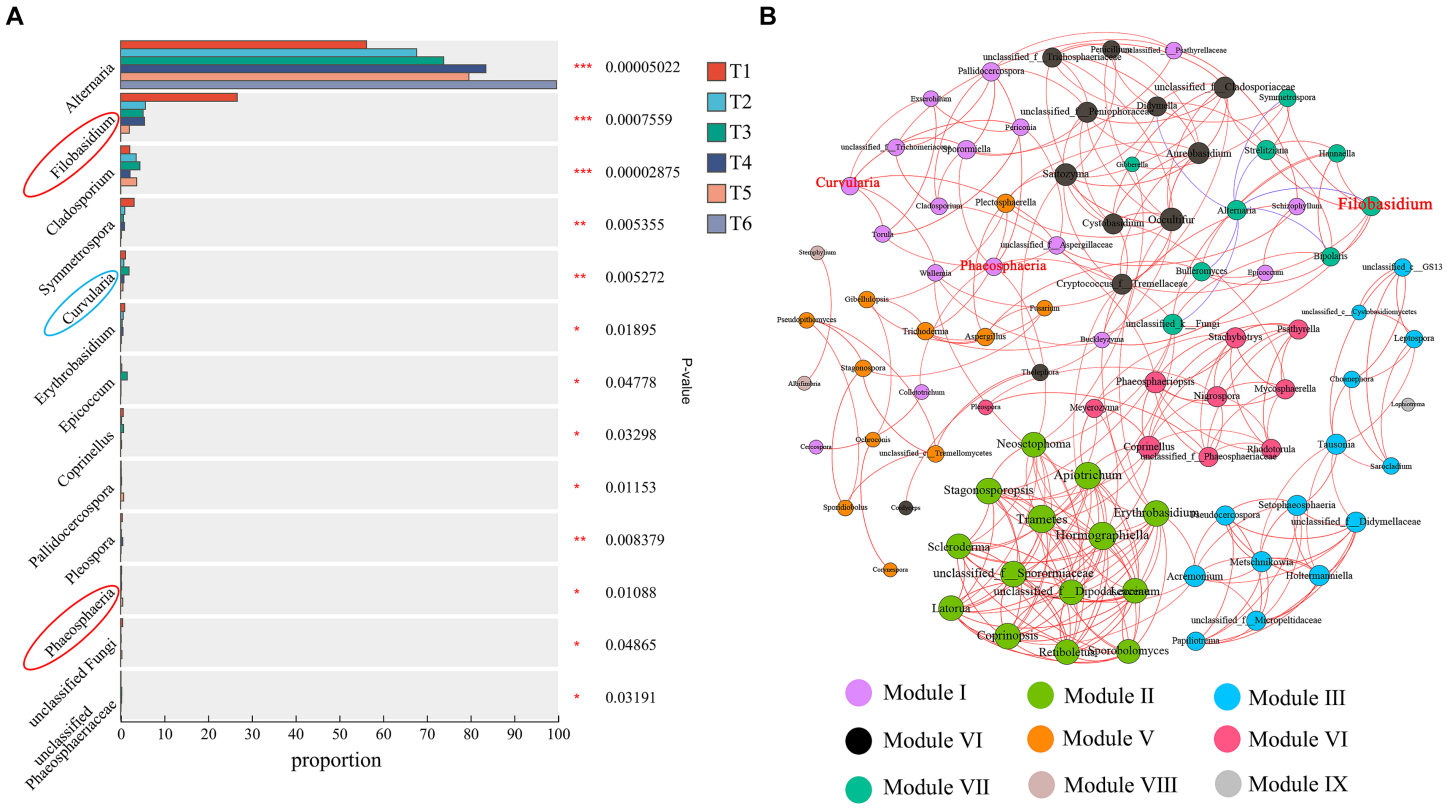
Analysis of variance **(A)** and co-occurrence network analysis of fungal abundance **(B)** during the AC process. ^*^p < 0.05, ^**^p < 0.01, ^***^p < 0.001. Red circles indicate Undefined Saprotroph; blue circles indicate Plant Pathogen. The size of the nodes in a network graph is proportional to the number of connections. The blue line indicates a positive correlation and the red line indicates a negative correlation.

A differential analysis of fungal communities during the AC of cigar tobacco leaves was performed (Figure 7A). The abundances of 13 bacterial genera changed significantly (*p* < 0.05) during the AC process. The relative abundance of *Alternaria* increased gradually with AC and was significantly higher at the end of AC than at both T1 and T2. *Filobasidium*, *Cladosporium*, *Symmetrospora*, *Curvularia*, *Erythrobasidium*, *Epicoccum*, *Coprinellus*, *Pallidocercospora*, *Pleospora*, *Phaeosphaeria*, unclassified Fungi, and unclassified *Phaeosphaeriaceae* decreased in abundance at the end of AC. These results further suggest that AC alters the relative abundance of fungal communities in cigar tobacco leaves. *Filobasidium* and *Phaeosphaeria* were highly abundant genera in Undefined Saprotroph (94.28%) and *Curvularia* was a highly abundant genus in Plant Pathogen (57.51%). The results further indicate that the Undefined Saprotroph (mainly *Filobasidium* and *Phaeosphaeria*) and Plant Pathogen (mainly *Curvularia*) groups are susceptible to the AC environment.

A network analysis of all fungal genera in the AC process showed that there were 90 nodes and 301 edges in the network (Figure 7B). Among them, 294 edges indicated positive correlations, while all negative correlations were from *Alternaria*, indicating that the fungi in tobacco were mainly positively correlated, resulting in strong synergistic interactions between fungal genera to cope with environmental changes during the AC process. The modularity index was 0.702, indicating a typical modular structure of the fungal interactions. The fungi were divided into nine modules during the AC process, while *Filobasidium*, *Phaeosphaeria,* and *Curvularia* (red font on the node), which were more variable in Undefined Saprotroph and Plant Pathogen, belonged to two modules. These three genera were more variable during AC, as they were probably influenced by fungi within the modules they belonged to.

## 4 Discussion

Unlike field conditions during the growing season, where tobacco plants receive moisture from the environment, the AC process of cigar tobacco leaves occurs without any external water supply. Consequently, the leaves undergo a natural AC process during which water is lost.

At the end of the AC process, the protein and starch contents of the leaves are lower, which improves the sensory comfort of the tobacco (Lin et al., 2020). As a vital component of cellular metabolism, water plays a crucial role in certain metabolic processes. Within a certain range, the amount of water directly affects the cell metabolic intensity (Saragovi et al., 2022). When harvested, tobacco leaves have a high moisture content, allowing them to undergo self-metabolism during the early stages of AC (Ai et al., 2010; Yamaguchi et al., 2013). Saprophytic fungi also decompose large molecular substances, such as proteins and starch (Chen Y. et al., 2020). Starch degradation generates sugars; however, the water-soluble sugar content decreases continually, which may be due to the consumption of sugar substances by microorganisms on the tobacco leaves.

During the AC process, the fungal phyla detected were mainly Ascomycota and Basidiomycota, which is consistent with the findings of Zhang et al. (2023). During AC, the water-soluble sugar (acidic substances) of tobacco leaves decreased and the nicotine (alkaline substances) did not change much; therefore, the sugar-nicotine ratio of tobacco leaves was always in a decreasing trend during AC, which resulted in a gradual increase in pH in the tobacco leaves. Higher pH can increase the abundance of ascomycetes, whereas stramenopiles are affected by pH, and their abundance decreases as pH increases (Sui et al., 2022). In addition, the efficient utilization of nutrients by ascomycetes leads to their higher abundance (Chen et al., 2017).

The AC of cigar tobacco leaves involves water loss and drying, which can adversely affect the fungus (Staddon et al., 2003; Liu H. et al., 2021; Liu F. et al., 2021). *Alternaria* in *Ascomycota* is able to regulate the contents of the phytohormone salicylic acid, which facilitates the resistance of the plant to environmental stress (Mishra et al., 2018), which leads to a gradual increase in Alternaria during the AC process. This may also explain why Alternaria is negatively correlated with other fungi in the fungal co-occurrence network diagram. Melanin produced by *Alternaria* also has anti-pathogenic functions (Singh et al., 2021), which may be another reason why there are fewer pathotrophs. Additionally, *Alternaria* pathogenicity is related to the production of melanin, which is formed by polyphenols (Singh et al., 2021). Unlike other agricultural products, cigar tobacco leaves must turn brown or even black-brown after AC, and this color is formed by polyphenols (Zhao et al., 2022). This may explain why *Alternaria*, despite being a pathogenic fungus, is highly abundant in cigar tobacco leaves and not categorized as “highly probable” or “probable” in functional groups. These results indicate that fungal abundance is affected by changes in substances and moisture content during the tobacco leaf AC process. However, the diversity and abundance index of fungi did not change significantly before the dry gluten stage, probably because the moisture content and pH in the tobacco leaves had not yet reached critical values that affect fungal community structure (Ge et al., 2022).

Fungi, as important microorganisms in nature, mainly participate in the degradation of macromolecular organic matter during plant wilting, especially saprotrophs, which are the main decomposers of dead or aging plants, and play an important role in the decomposition of macromolecular organic matter and nutrient cycling (Chen Q. L. et al., 2020; Nelson et al., 2020; Kang et al., 2023). In addition, higher total carbon and total nitrogen contribute to saprotroph accumulation (Kang et al., 2023; Wang et al., 2023). The AC of cigar tobacco is a process of secondary aging, or rather, the process of water loss and withering of tobacco leaves, and saprotrophs obtain nutrition by degrading dead host cells. Therefore, saprotrophs have the highest proportion during the AC process. Furthermore, saprotrophs release substrates required for aroma substance formation (Lu et al., 2020) and survival of other microbes in tobacco leaves by degrading macromolecular organic matter, such as starch and protein. This results in a decrease in total carbon and total nitrogen in tobacco leaves as AC proceeds, which in turn reduces saprotroph abundance. In addition, an increase in the internal environmental pH of the tobacco leaves during AC reduces saprotroph abundance (Wu et al., 2022). This could also be the reason why saprotrophs exhibited a positive correlation with quality indicators in tobacco leaves. This also explains the decreasing trend of *Filobasidium*, which has the highest percentage of saprotrophs, during the AC process, after tobacco leaves are harvested.

*Curvularia* is the main pathotroph in tobacco leaves and is detrimental to crop quality (Fajola, 1979). Saprotrophs have antagonistic effects with pathotrophs (Yu et al., 2022), which may be the reason why the main pathotrophs in tobacco leaves, *Curvularia*, increased with a decrease in saprotrophs, *Filobasidium*, in the early stage of the AC process. *Curvularia* reached its maximum in the yellowing stage, which is also the stage of massive water loss in tobacco leaves. The stress-resistant substances in the leaves decrease and the resistance of the leaves decreases, resulting in a higher proportion of pathotrophs. Subsequently, *Curvularia* exhibit a decreasing trend, which is attributed to the continued loss of water from the tobacco leaves, and the low-moisture environment in turn suppresses *Curvularia* (Wang et al., 2014). We observed that symbiotroph abundance was consistently low during AC, which may be due to the tobacco leaves consistently losing water during AC, which corresponds to an extreme drought, and the environment is not favorable for symbiotroph growth (Wu et al., 2022).

## 5 Conclusion

This study investigated the dynamic changes in fungal communities and their functions during the AC process of cigar tobacco leaves. We found that the changes in fungal communities during the AC process were dynamic and responsive to changes in the AC environment, with *Alternaria* abundance remaining consistently high. Throughout the AC process, there was a predominance of saprophytic fungi that can degrade macromolecules in tobacco leaves. However, as the moisture content and carbon–nitrogen substances (excluding nicotine) in the leaves decreased during AC, the nutritional resources available for the fungi decreased, resulting in a reduction in the number of saprophytic fungi, which reached their lowest levels by the end of the AC process. This study provides theoretical guidance for the relationship between fungi and tobacco quality during the AC of cigar tobacco leaves. As a next step, we could modulate the process of AC, thus utilizing saprotrophs to degrade macromolecules in tobacco. Alternatively, a saprotroph could be selected for use as an exogenous additive. These findings can also be expanded to other crops, such as tea. The mechanisms of interactions between quality indicators and saprotrophs in tobacco leaves could also be explored further.

## Data availability statement

The datasets presented in this study can be found in online repositories. The names of the repository/repositories and accession number(s) can be found at: https://www.ncbi.nlm.nih.gov/, PRJNA1054308.

## Author contributions

SZ: Data curation, Formal analysis, Investigation, Methodology, Writing – original draft. YLi: Investigation, Supervision, Writing – review & editing. FL: Investigation, Supervision, Writing – review & editing. ZS: Data curation, Investigation, Software, Supervision, Writing – review & editing. WY: Writing – review & editing. YLe: Writing – review & editing. PT: Data curation, Writing – review & editing. MZ: Funding acquisition, Investigation, Resources, Supervision, Writing – review & editing.

## Footnotes

^1^ cloud.majorbio.com

^2^ http://www.funguild.org/

^3^ http://www.biomicroclass.com/
